# Adult Colonic Intussusception: A Focused Review of Diagnostic and Management Strategies

**DOI:** 10.3390/medicina62040747

**Published:** 2026-04-13

**Authors:** Tudor-Alexandru Popoiu, Cicerone Catalin Grigorescu, Stelian Pantea, Dan Brebu, Mircea Selaru

**Affiliations:** 1 Department of Functional Sciences, Medical Informatics and Biostatistics Discipline, “Victor Babes” University of Medicine and Pharmacy Timisoara, 300041 Timisoara, Romania; tudor.popoiu@umft.ro; 2 Doctoral School, Faculty of Medicine, “Victor Babes” University of Medicine and Pharmacy Timisoara, 300041 Timisoara, Romania; 3 IIIrd Surgery Clinic of “Pius Brinzeu”, County Emergency Clinical Hospital Timisoara, 300723 Timisoara, Romania; pantea.stelian@umft.ro (S.P.); selaru.mircea@umft.ro (M.S.); 4 Department of Anatomy, Faculty of General Medicine, Vasile Goldiș Western University of Arad, 310025 Arad, Romania; 5 X Department of General Surgery, “Victor Babes” University of Medicine and Pharmacy Timisoara, 300041 Timisoara, Romania; brebu.dan@umft.ro; 6 Discipline of Surgical Emergencies, Department of Surgery III, “Victor Babes” University of Medicine and Pharmacy Timisoara, 300041 Timisoara, Romania; 7 IInd Surgery Clinic, Timișoara Emergency County Hospital, 300723 Timisoara, Romania

**Keywords:** adult intussusception, rectal prolapse, colorectal malignancy, neoplastic lead point, bowel obstruction, villous adenoma

## Abstract

Adult intussusception is a rare cause of intestinal obstruction that differs markedly from the pediatric form in etiology, clinical presentation, and management. In contrast to predominantly idiopathic pediatric cases, adult intussusception is usually associated with an underlying structural lesion, particularly malignancy in colonic involvement. This narrative review summarizes current evidence regarding the epidemiology, etiologic spectrum, clinical features, diagnostic evaluation, and management of adult colo-colic and sigmoido-rectal intussusception. Clinical presentation is often nonspecific, and distal variants may mimic rectal prolapse or large bowel obstruction, contributing to delayed diagnosis. Contrast-enhanced computed tomography represents the diagnostic modality of choice, enabling the identification of lead points and associated complications. Surgical resection remains the cornerstone of treatment due to the high risk of malignancy, while nonoperative management is reserved for carefully selected cases. Improved recognition of atypical presentations and individualized, imaging-guided management are essential to optimize outcomes in this uncommon but clinically significant condition.

## 1. Introduction

Intussusception is classically regarded as a disease of infancy and early childhood, where it most commonly presents as an idiopathic condition amenable to nonoperative reduction. In adults, however, intussusception is a rare and diagnostically challenging entity, accounting for a small fraction of bowel obstructions and frequently associated with an underlying structural lesion [1]. Unlike pediatric cases, adult intussusception often follows a subacute or chronic course, with nonspecific symptoms that may delay diagnosis and definitive management [2].

Among adult presentations, colonic intussusception carries particular clinical significance due to its strong association with malignant lead points and the consequent implications for surgical strategy [3]. Within this group, sigmoido-rectal intussusception represents an especially uncommon and underrecognized variant. Its rarity, combined with its propensity to present with anorectal symptoms or an externally prolapsing mass, can lead to diagnostic confusion with more common conditions such as primary rectal prolapse. Failure to recognize this distinction may result in inappropriate management and potential oncologic compromise [4]. Advances in cross-sectional imaging, particularly computed tomography (CT), have improved preoperative diagnostic accuracy. A substantial proportion of adult cases continue to be identified only intraoperatively [5]. Moreover, consensus regarding optimal management especially the role of reduction versus en bloc resection in colonic disease—remains rooted in observational data rather than high-level evidence, underscoring the importance of detailed case-based analyses [6,7].

Although this work is presented as a narrative review, a structured literature search was performed to ensure comprehensive coverage of relevant studies. Electronic databases including PubMed, Scopus, and Google Scholar were searched for articles published up to January 2026. The search strategy combined terms such as “adult intussusception”, “colonic intussusception”, “colo-colic”, “sigmoido-rectal”, and “rectal prolapse”. Studies involving adult patients were prioritized, with particular focus on colonic and colorectal involvement. In addition, selected literature on pediatric intussusception and small bowel disease was included to provide broader clinical and pathophysiological context. Reference lists of relevant articles were also screened to identify additional reports. Emphasis was placed on studies addressing epidemiology, etiology, clinical presentation, imaging, and management.

The available evidence on adult intussusception is predominantly derived from retrospective case series, small institutional cohorts, and systematic reviews, with substantial heterogeneity in study design, patient populations, and reported outcomes. As such, many of the quantitative estimates presented in this review—including symptom frequencies, imaging performance, and prognostic indicators—should be interpreted as approximate ranges rather than pooled or standardized measures. Where possible, distinctions between data derived from individual studies and broader literature trends have been highlighted.

This narrative review synthesizes the current literature on adult colo-colic and sigmoido-rectal intussusception, with particular emphasis on epidemiology, etiologic patterns, clinical presentation, diagnostic strategies, and management principles. Despite increasing recognition through cross-sectional imaging, adult intussusception remains a diagnostically challenging entity, and clear, practical guidance—particularly for distal colonic and prolapsing presentations—is limited. Although broader adult and pediatric data are referenced where relevant, the primary focus of this review is on colonic and sigmoido-rectal intussusception, given their distinct malignant potential and surgical implications. This review aims to highlight key diagnostic pitfalls, the central role of CT imaging in identifying clinically significant disease, and the implications of malignancy risk for surgical decision-making. By integrating available evidence, we seek to provide a clinically applicable framework to support surgeons, gastroenterologists, and emergency physicians in the evaluation and management of atypical large bowel obstruction and anorectal presentations.

## 2. Epidemiologic and Clinical Context

Intussusception, defined as telescoping of a proximal bowel segment into the lumen of an adjacent distal segment, is predominantly a pediatric disease. In adults it is distinctly uncommon, accounting for approximately 5% of all intussusception cases and roughly 1% of intestinal obstructions [4,7,8]. Adult intussusception differs fundamentally from pediatric disease in its epidemiologic profile, etiologic spectrum, and management: whereas most pediatric cases are idiopathic and often reducible by non-operative means, the vast majority of adult cases are secondary to an underlying structural lesion, and operative treatment is usually required [4,9,10]. Across adult series, small-bowel involvement predominates, with colonic, colo-colic, and colo-rectal intussusceptions comprising roughly one-quarter to one-third of cases. Within this colonic subset, distal involvement of the sigmoid colon and rectum is particularly rare, such that “sigmoido-rectal intussusception” rarely appears as a discrete category in larger case series [4,7,8,10,11]. Nonetheless, scattered case reports and small series demonstrate that the sigmoid colon may invaginate into the rectum and even prolapse through the anal canal, producing a clinical picture that overlaps with, and is sometimes misdiagnosed as, rectal prolapse [12,13,14].

## 3. Etiologic Spectrum and Malignancy Burden

A defining feature of adult intussusception is the high prevalence of organic lead points. Across pooled analyses and institutional series, only 8–20% of adult cases are considered idiopathic, with the remainder attributable to benign or malignant structural lesions such as neoplasms, inflammatory disease, postoperative changes, Meckel’s diverticulum [7,8,10,11,15] or endometriosis [16]. This is particularly true in the colon and rectum: all major reviews and series concur that adult colonic/colo-rectal intussusception is overwhelmingly structural and frequently malignant [7,8,10]. It should be noted that many published series report mixed adult intussusception cohorts, predominantly involving small bowel disease. However, colonic and sigmoido-rectal intussusceptions differ significantly, with a markedly higher likelihood of malignant lead points. Colonic lead lesions are predominantly adenocarcinomas, with less frequent contributions from lymphoma, metastatic deposits, and submucosal tumors, while benign colonic lesions include adenomas, lipomas, and inflammatory or hamartomatous polyps [7,8,10,11,12]. Mohamed et al. highlights a sigmoido-rectal intussusception caused by a large submucosal lipoma, illustrating that substantially sized benign lesions in the sigmoid colon can precipitate complete obstruction and telescoping into the rectum, even in the absence of the classic acute triad, which will be discussed further [17].

The benign causes of colo-colic intussusception include colonic lipomas (the most common benign cause), which typically range in size from 4 to 16 cm with an average of 7 cm. Other benign etiologies are less frequent and include adenomatous polyps (particularly in younger adults), stromal tumors (gastrointestinal stromal tumors), inflammatory polyps [18,19,20], and appendiceal mucoceles [21].

Additional pathological lead points documented in the literature include Crohn disease granulomas, tuberculosis, submucosal hemorrhages from unregulated anticoagulation, intestinal ulcers from Yersinia infection, Henoch–Schönlein purpura, and Peutz–Jegers Syndrome [22,23,24]. Geographic variation in etiology has been documented, with “Ibadan intussusception” or “tropical intussusception” in central and western Africa most commonly presenting as ceco-colic intussusception, with etiologies attributed to fiber content of diet, dietary habits, genetics, and gut microbiome composition [25].

## 4. Clinical Presentation, Symptoms, and Laboratory Findings

The clinical presentation of adult intussusception is notably different from the pediatric presentation and tends to be nonspecific and variable (as seen in Table 1). There are notable differences as well when it comes to small intestines versus colonic intussusception (as seen in Table 2). The classic triad of abdominal pain, palpable abdominal mass, and blood-positive stools is rarely encountered in adults [11,26]. The most frequent symptoms include abdominal pain in 96.42% of patients as reported in individual series, typically colicky and intermittent in early phases, and becoming more constant as obstruction or ischemia progresses, followed by nausea and vomiting in 71.42% of cases, which may range from bilious to fecaloid. Other reported symptoms include abdominal distention, constipation, diarrhea, and bloody stools ranging from occult blood loss to overt hematochezia, is frequently observed when the lead point is neoplastic or ulcerated [23,27]. In some cases, spontaneous reduction may occur, resulting in temporary symptom relief and potentially delaying diagnosis [28]. The symptom duration varies considerably. In one series of 28 adult intussusception patients, the median overall symptom duration from onset to diagnosis was 18 days (range: 1–365 days), with 17.86% presenting with acute symptoms, 17.86% with subacute symptoms, and 64.28% with chronic symptoms. This variability reflects the heterogeneous presentation patterns and the diagnostic delay that often accompanies this condition [27]. The presence of weight loss may serve as a clinical indicator suggesting serious underlying pathology and should alert clinicians to suspect malignancy. Bloody diarrhea or rectal bleeding, when present, suggests mucosal sloughing from necrotic tissue, indicating more advanced disease [11].

Physical examination findings commonly include abdominal distension and tenderness, abdominal tympany may be marked, while peritoneal signs are typically absent unless ischemia or perforation has supervened [23]. However, the physical examination is often nonspecific and may not distinguish intussusception from other causes of acute or subacute abdominal pain [29].

Adult sigmoido-rectal intussusception presents along a spectrum that bridges two traditionally distinct clinical domains: large-bowel obstruction and anorectal prolapse. As in adult intussusception more generally, symptoms are frequently nonspecific and often reflect the underlying lead lesion and degree of obstruction rather than the telescoping process per se [8,13]. In the distal colon and rectum, however, these features are overlaid by pelvic and outlet-type symptoms that can closely mimic primary rectal prolapse [12,13].

On physical and digital rectal examination, the presence of a focal leading lesion on the prolapsed segment, an eccentric or irregular configuration with a discernible space between the prolapsing bowel and the native anal canal wall, and the absence of uniform concentric folds should raise strong suspicion for sigmoido-rectal intussusception rather than primary rectal prolapse, particularly when accompanied by bleeding or signs of proximal obstruction [7,12].

### 4.1. Obstructive Presentations

In some patients, the intussuscepted sigmoid colon functions as a mechanical plug within the rectum, producing a clinical picture akin to distal colonic obstruction. In Du et al.’s report of rectosigmoid (sigmoido-anal) intussusception pronounced dilation of the proximal colon and a distended caecum, illustrates the capacity of a distal rectosigmoid intussusception to create a functional “closed-loop” obstruction [11,12,30]. Occlusive presentations may occur even with frank fecal vomiting and rectal bleeding, as described by Kada et al. [28].

### 4.2. Prolapse-like and Outlet-Type Presentations

A distinctive feature of sigmoido-rectal and recto-sigmoidal intussusception is its propensity to present with a prolapsing anorectal mass, sometimes as the dominant or sole obvious abnormality. Du et al. described a long-segment sigmoid intussusception that prolapsed through the anal canal and was initially indistinguishable from full-thickness rectal prolapse on inspection [30], differences that are highlighted in Table 3. Ganesan et al. likewise reported a case of sigmoido-rectal intussusception with a protruding mass at the anal verge, with progression over 3–4 months, being continually self-reduced during this time [31]. Mahmood et al. described colo-colic intussusception prolapsing through the anus due to a villous adenoma, initially misinterpreted as benign rectal prolapse, highlighting the risk of delayed oncologic diagnosis [32]. Similarly, Sseruwagi and Lewis reported rectal prolapse associated with intussusception caused by rectal adenocarcinoma, emphasizing the diagnostic difficulty and the importance of prompt surgical exploration [33]. Filiberto et al. further demonstrated sigmoido-rectal intussusception secondary to obstructing sigmoid carcinoma presenting as full-thickness rectal prolapse, with cross-sectional imaging identifying the malignant lead point and guiding definitive resection [34]. Collectively, these reports underscore that apparent rectal prolapse in adults—particularly when acute, irreducible, or associated with obstructive symptoms—should raise suspicion for underlying intussusception and malignancy, warranting thorough imaging and timely surgical management.

### 4.3. Particular Presentations

While neoplastic lead points can result in sigmoido-rectal or colo-anal intussusception with external rectal prolapse, post-endoscopic inflammatory changes represent a separate non-prolapsing entity. Rienecker and Rawther reported a rare case of colo-colic intussusception following routine colonoscopy with cold-snare polypectomy in a 58-year-old woman who presented abdominal pain, rectal bleeding, and fever 15 h post-procedure. CT demonstrated the characteristic “target sign,” confirming intussusception, while repeat colonoscopy showed erythematous, edematous mucosa consistent with spontaneous resolution, allowing conservative management and avoidance of surgery. The authors highlight colonoscopic manipulation and post-polypectomy mucosal edema as potential precipitating factors and emphasize the importance of maintaining a high index of suspicion for this uncommon but significant post-procedural complication [35]. Moon et al. described colo-colonic intussusception following electrocoagulation polypectomy, attributed to bowel wall edema associated with post-polypectomy electrocoagulation syndrome, without rectal prolapse or malignant lead point, and successfully managed conservatively [36]. Gültekin et al. reported a rare case of idiopathic, non-obstructive colo-colonic intussusception in a 41-year-old man presenting with nonspecific abdominal pain, in whom cross-sectional imaging revealed a “bowel-within-bowel” configuration without an identifiable lead point [37]. Colonoscopy excluded malignancy, and laparoscopic resection confirmed intussusception with only nonspecific inflammatory changes on histology, highlighting that adult colo-colonic intussusception may occasionally occur in the absence of structural pathology and present with subtle clinical features. A rare presentation was described in a 37-year-old woman with acute abdominal pain and distension following several days of intermittent symptoms. CT imaging revealed extensive colonic intussusception extending from the ascending colon to the splenic flexure, with a suspected mass as the lead point. Surgical exploration with right hemicolectomy confirmed a plasmablastic neoplasm involving the ileocecal valve, highlighting that atypical hematologic malignancies may rarely present as colonic intussusception [38].

### 4.4. Laboratory Investigations

Laboratory findings in adult intussusception are nonspecific and primarily reflect disease severity rather than diagnosis: mild anemia may be present in cases with ulcerated adenomas or carcinomas due to chronic blood loss, inflammatory markers typically remain normal unless ischemia or perforation develops, electrolyte and renal abnormalities correlate with the degree of obstruction and vomiting, and tumor markers such as carcinoembryonic antigen lack validated diagnostic value in this setting [10,11,39].

### 4.5. Differential Diagnosis

Given the nonspecific presentation of adult intussusception, it must be considered in the differential diagnosis of acute and subacute abdominal pain. The differential diagnosis in emergency medicine includes gastric volvulus, internal hernias, colonic volvulus, other causes of bowel obstruction, acute gastroenteritis, appendicitis, and abdominal trauma. The broad differential and the frequent delay in diagnosis emphasize the importance of maintaining a high index of clinical suspicion, particularly in patients presenting with intermittent or chronic abdominal pain and nonspecific gastrointestinal symptoms [40].

## 5. Diagnostic Imaging

Diagnosis of adult intussusception, particularly colo-colic forms, presents a significant clinical challenge due to the nonspecific presentation and the relatively rare frequency of the condition. Multiple imaging modalities have been evaluated.

Computed Tomography has emerged as the imaging modality of choice. Abdominal CT scan demonstrates reported diagnostic accuracy ranging from 58% to 100%, depending on the series reviewed and the expertise of the radiologist. The characteristic finding is the pathognomonic “bowel-within-bowel” appearance, often containing mesenteric fat and blood vessels. Three radiological patterns are recognized: the “target” or “bull’s-eye sign” is formed by a thickened intestinal wall and mesentery within the lumen in axial projections; the “sausage-shaped” pattern appears in longitudinal projections with alternating areas of low and high attenuation related to bowel wall, mesenteric fat, fluid, and contrast; and the “reniform” pattern appears as a bilobed mass with central low attenuation and peripheral higher density, thought to result from ischemic thickening of the intussusceptum’s bowel wall [7,41,42]. CT imaging provides critical additional information beyond diagnosis, including identification of the lead point in 94.4% of patients where a structural cause exists, precise delineation of involved segments, assessment of bowel wall thickness and enhancement characteristics, and identification of complications including bowel wall ischemia, perforation, and peritoneal findings. The concordance between pathology results and radiological diagnostic suspicion can reach 94.2% [7,43].

Ultrasonography is useful in selected populations, particularly in pediatric patients and those without obesity or significant bowel gas. The characteristic images include the “target sign” or “doughnut sign” in cross-section and the “pseudokidney sign” in longitudinal section, corresponding to the intussusceptum and surrounding hyperechoic mesenteric fat within the intussuscipiens. Reported sensitivity ranges from 97.9% to 98.5% with specificity of 97.8% to 100%. However, technical limitations in adults include obesity and bloating, which reduce image quality and diagnostic accuracy [44,45,46].

Plain radiography has a limited but specific role. It may reveal indirect signs of intussusception such as air-fluid levels indicating intestinal obstruction, or the rare “crescent sign” (gas between the intussusceptum and intussuscipiens), though this sign is rarely identified in practice. More importantly, plain radiography can identify complications such as pneumoperitoneum or high-grade bowel obstruction [45].

Although MRI is generally not recommended for the evaluation of adult intussusception and is more commonly used in small bowel involvement [47], pelvic MRI was reported in one case shortly after reduction of the prolapsed intussusception [31].

Colonoscopy can serve both diagnostic and potentially therapeutic purposes in selected cases, allowing for biopsy confirmation of the underlying lesion and potentially enabling polypectomy for adenomatous polyps. However, colonoscopy cannot reliably determine if a lesion is benign or malignant based on endoscopic appearance alone, particularly in cases of mucosal ulceration or necrosis [13,18,20].

Although the widespread use of CT has increased preoperative diagnostic accuracy to approximately 70–81%, a substantial proportion of adult intussusceptions remain unrecognized prior to surgery; notably, colonic involvement is a strong predictor of surgical relevance, as colonic intussusceptions account for up to 63% of lesions requiring operative treatment, whereas only about 5–10% of predominantly entero-enteric intussusceptions necessitate surgical intervention and are frequently self-limiting [48].

## 6. Management

We have already established clear differences between adult and pediatric forms of intusussception, and the management of colo-colic/colo-rectal intussusception in adults is no exception from this rule and is guided by multiple clinical and pathological factors. Importantly, management principles differ between small bowel and colonic intussusception. While reduction may be considered in selected entero-enteric cases, colonic involvement generally warrants primary oncologic resection due to the higher risk of malignancy. Surgical treatment represents the cornerstone of management, whether performed via minimally invasive or open approaches, given the high likelihood of an underlying malignant lead point. Accordingly, oncologic resection with negative margins (R0 resection) is generally recommended to optimize long-term outcomes. Nonoperative management has been described in selected cases, particularly in transient and uncomplicated intussusceptions, and will be briefly discussed. Importantly, management strategies differ substantially between small-bowel and colonic intussusception; the following discussion focuses on colonic disease, where oncologic resection is generally recommended.

### 6.1. Surgical Management

The prevailing treatment for adult intussusception remains surgical intervention. However, controversy exists regarding the optimal surgical approach, specifically whether to attempt intraoperative reduction or to perform en bloc resection without reduction.

The “en bloc resection without reduction” strategy has been advocated by many surgeons as the preferred approach due to the high likelihood of underlying malignancy and the theoretical risk of tumor cell dissemination or upstaging if the lesion is manipulated. This approach involves complete removal of the intussuscepted segment in one piece along with appropriate margins, particularly for potentially malignant lesions [11,49,50,51].

The extent of resection depends on the underlying pathology and the length of invaginated bowel. Common procedures include right hemicolectomy (for ceco-colic intussusception), left hemicolectomy (for left-sided lesions), and subtotal colectomy with ileorectal anastomosis (for extensive invagination). Segmental resection may be possible when benign etiology is confirmed, such as for isolated lipomas [19,52]. Primary anastomosis is preferred when deemed technically and clinically safe.

#### Minimally Invasive Approaches

Laparoscopic surgery has been increasingly employed for both diagnostic and therapeutic purposes. Laparoscopy allows for initial exploration and assessment, and can facilitate reduction with forceps separation of intestinal segments. However, laparoscopy is not universally applied in intussusception cases, largely because these conditions often present as emergencies and laparoscopic approaches cannot guarantee the safety of surgery, particularly when intestinal anastomosis is needed [12,14,53]. Advances in laparoscopic techniques, minimally invasive surgery is increasingly used and appears feasible and safe, offering advantages such as reduced pain and faster recovery [54,55]. Laparoscopy also provides valuable diagnostic insight and may avoid unnecessary laparotomy, although clear consensus regarding its safety and oncologic adequacy is still lacking [56]. Current evidence is limited to small series and case reports.

### 6.2. Nonoperative Management

While therapeutic enemas and colonoscopic reduction represent the mainstay of treatment in pediatric intussusception, apart from the ileo-ileal situations, these approaches are far less commonly used in adults because of the high likelihood of an underlying malignant lead point.

Conservative management with bowel rest, intravenous fluid support, pain management, and close clinical monitoring may be appropriate for selected cases, particularly nonsurgical or transient intussusceptions without evidence of obstruction or ischemia. However, transient non-obstructing intussusceptions without lead points are more common in small bowel disease (particularly associated with celiac disease or Crohn disease) than in colonic intussusception [11,26,29].

### 6.3. Practical Imaging-Guided Management Algorithm

**Figure 1:**
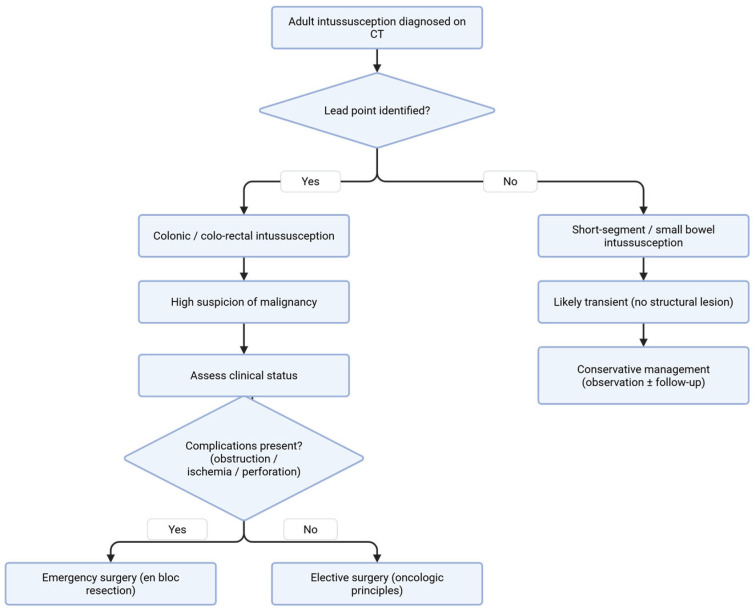
Imaging-Guided Management Algorithm for Adult Intussusception (Created in BioRender. Popoiu, T.A. (2026) https://BioRender.com/pqlnkvf (accessed on 13 March 2026)).

Based on the available evidence, a pragmatic, imaging-guided approach can facilitate clinical decision-making in adult intussusception (Figure 1).

The initial step is differentiation between transient, short-segment intussusception without a lead point, most commonly seen in small bowel disease, and true intussusception with an identifiable structural lesion, particularly in colonic or sigmoido-rectal cases. In patients with transient intussusception, characterized on CT by short segment involvement, absence of bowel obstruction, lack of a lead point, and preserved bowel wall enhancement, conservative management with clinical observation is generally appropriate [8].

In contrast, patients with colonic or colo-rectal intussusception, especially when associated with a visible lead point, should be considered at high risk for malignancy and evaluated for surgical intervention [57,58].

Further stratification should be based on the patient’s clinical status. Patients presenting with obstruction, ischemia, or perforation require urgent surgical exploration. In contrast, stable patients without signs of complications are best managed with elective oncologic resection. In cases presenting with a prolapsing anorectal mass, a high index of suspicion for sigmoido-rectal intussusception is essential, and cross-sectional imaging should guide prompt surgical management [58,59].

This framework highlights the central role of CT imaging in guiding both diagnosis and treatment strategy, while emphasizing that colonic intussusception should generally be managed with an oncologic surgical approach. To date, no standardized management algorithm for adult intussusception has been universally adopted; this framework represents a pragmatic synthesis of current evidence to facilitate clinical decision-making. These recommendations are primarily supported by retrospective series and expert consensus, as prospective comparative studies are lacking.

### 6.4. Surgical Complications and Outcomes

The operative morbidity associated with surgical management varies in reported series. One report documented perioperative 30-day mortality of 8% and postoperative morbidity of 32% in a cohort of 28 adult intussusception patients, with complications including abdominal sepsis, hospital-acquired pneumonia, septic shock, urinary tract infection, and acute kidney injury. Another series reported postoperative morbidity rates of 22.1%, with surgical site infection being the most common complication, and overall mortality of 5.2% [60].

The mortality and morbidity are significantly influenced by age. A recent large-scale analysis revealed that elderly patients with intussusception experienced mortality rates 28 times higher than adults (2.8% vs. 0.1%), demonstrating advanced age as a critical predictor of mortality. In operatively managed elderly patients, higher frailty scores were associated with 34.0% greater mortality odds, while female sex reduced odds by 69.8% [61].

### 6.5. Predictive Factors for Outcomes

Several factors influence the need for bowel resection and development of ischemic complications. Delayed surgical intervention substantially increases mortality: each day of delayed surgery raised mortality odds by 26.7% in adult patients. Other unfavorable prognostic indicators include longer hospital length of stay, severe comorbidities (including coagulopathy, liver disease, neurological disorders, and weight loss), and signs of advanced ischemia such as red currant jelly stool [28,61].

Inflammatory biomarkers have been investigated for their utility in predicting intestinal necrosis and the need for resection. The combination of lymphocyte count with C-reactive protein levels (LCR index) demonstrated sensitivity of 0.82 and specificity of 0.80 for predicting strangulation, suggesting potential clinical utility for preoperative risk stratification [62].

### 6.6. Recurrence

Recurrence of intussusception after treatment depends on the surgical approach employed. In one series examining outcomes, there was no recurrence in the resection group, while one recurrence was noted in the reduction-only group on 3-month follow-up, requiring readmission and resection of small bowel with primary anastomosis. These findings suggest that definitive resection, as opposed to reduction alone, may be protective against recurrence [63].

### 6.7. Complications of the Disease

Intussusception itself can lead to serious complications if not promptly recognized and managed. Bowel ischemia and necrosis represent the most significant complications, resulting from progressive vascular compromise. The ischemic intestine can progress to perforation, leading to peritonitis and sepsis, which are life-threatening conditions. These serious complications underscore the need for early diagnosis and timely intervention [60,62].

The available evidence on adult intussusception is largely derived from retrospective case series, small institutional cohorts, and isolated case reports, with considerable heterogeneity in patient populations, anatomical subtypes, and management strategies. Consequently, many of the recommendations discussed in this review—particularly regarding reduction versus en bloc resection, the role of laparoscopy, and the use of conservative management—are based on observational data and expert opinion rather than high-level comparative studies. This limitation is especially relevant in colonic and sigmoido-rectal intussusception, where clinical decision-making must balance the risk of malignancy against procedural considerations. These factors highlight the need for cautious interpretation of the literature and underscore the importance of individualized, imaging-guided management. To provide a structured overview of the available evidence, we summarized the main findings from cohort studies and case series on adult intussusception (Table 4). These studies consistently demonstrate that adult intussusception presents with nonspecific symptoms, most commonly abdominal pain and features of bowel obstruction, and is frequently associated with an underlying structural lesion. Notably, colonic involvement, although less common than small-bowel disease, carries a substantially higher risk of malignancy and therefore has important implications for surgical management.

## 7. Conclusions

Adult intussusception remains a rare and diagnostically challenging condition, particularly in colonic and sigmoid-orectal forms, where the risk of an underlying structural lesion—often malignant—is substantial. Advances in cross-sectional imaging, especially computed tomography, have improved diagnostic accuracy and play a central role in guiding management. Surgical resection remains the cornerstone of treatment in colonic disease, reflecting the high likelihood of malignancy. However, current recommendations are largely based on retrospective series, small cohort studies, and case reports, with limited high-level evidence to support standardized management strategies. Consequently, clinical decision-making should be individualized, taking into account the anatomical location, presence of a lead point, suspicion of malignancy, and bowel viability. Further studies are needed to better define optimal diagnostic and therapeutic approaches in this uncommon but clinically significant condition.

## Figures and Tables

**Table 1:** Key Differences Between Pediatric and Adult Intussusception.

Feature	Pediatric Intussusception	Adult Intussusception
Incidence	Common	Rare (≈5%)
Etiology	Idiopathic (≈90%)	Organic lead point (80–92%)
Typical Location	Ileocolic	Small bowel > Colonic
Malignancy Risk	Rare	High (especially colonic)
Clinical Course	Acute	Subacute or chronic
Classic Triad	Common	Rare
Primary Imaging	Ultrasound	CT scan
Treatment	Nonoperative reduction	Surgical resection

**Table 2:** Key Features of Adult Intussusception with Emphasis on Colonic Involvement.

Feature	Small Bowel Intussusception (Adult)	Colonic/Sigmoido-Rectal Intussusception
Etiology	Often benign or transient, may lack lead point	Usually structural lead point
Malignancy Risk	Low to moderate	High, especially in colonic cases
Clinical Course	Often transient or intermittent	More likely to present with obstruction
Presentation	Abdominal pain, nonspecific	Obstruction, bleeding, or prolapse-like symptoms
Imaging Role	CT identifies transient vs. pathological	CT critical for detecting lead point
Management	Often conservative if no lead point	Surgical resection generally recommended

**Table 3:** Sigmoido-rectal Intussusception vs. Primary Rectal Prolapse.

Feature	Sigmoido-Rectal Intussusception	Primary Rectal Prolapse
Etiology	Structural lead point	Pelvic floor dysfunction
Onset	Acute or subacute	Chronic, progressive
Pain	Frequent	Usually minimal
Bleeding	Common	Occasional
Prolapsed Mass	Irregular, asymmetric	Symmetric, concentric folds
Leading Lesion	Often palpable	Absent
Digital Reduction	Often impossible	Usually possible
CT Findings	Bowel-within-bowel	Normal rectosigmoid anatomy
Oncologic Risk	Frequently associated	Not associated

**Table 4:** Summary of cohort studies and case series informing diagnosis and management of adult intussusception: This table summarizes available cohort studies and case series on adult intussusception. Across studies, clinical presentation is typically nonspecific, most commonly involving abdominal pain and features of bowel obstruction. A structural lead point is identified in the majority of cases, with malignancy rates ranging from approximately 20% to over 60%, particularly in colonic involvement. Computed tomography is consistently reported as the most reliable diagnostic modality. Surgical treatment remains the cornerstone of management, with oncologic resection preferred in colonic cases. The absence of idiopathic cases in several series further supports the predominance of underlying pathology in adult intussusception.

Study	Type	N	Colonic (%)	Malignancy (%)	Key Findings	Management
**Barris et al., 2025 [61]**	National cohort	4432	—	Up to 66% (colonic)	Nonspecific presentation; risk factors for mortality include age, frailty, delayed surgery	Surgery often required
**Lindor et al., 2012 [51]**	Retrospective cohort	148	—	~60% with lead point	Abdominal pain predominant; CT highly accurate	~50% surgical
**Kang et al., 2020 [56]**	Multicenter cohort	71	—	27% malignant	Laparoscopy feasible; faster recovery vs. open	Surgical management
**Rehman et al., 2010 [63]**	Case series	19	~10% (2/19)	~20% malignant	Obstructive presentation common; CT useful	Surgery in 95%
**Álvarez-Bautista et al. [27]**	Retrospective cohort	28	~21%	32%	Abdominal pain dominant (96%); heterogeneous presentation; colorectal cases uncommon	Surgery in all colonic cases
**Tarchouli et al. [11]**	Retrospective series	26	27%	35%	CT diagnostic ~81%; no idiopathic cases; high rate of structural lesions	Resection standard

## Data Availability

No new data were created or analyzed in this study.

## Abbreviations

The following abbreviations are used in this manuscript:

CT	Computed Tomography
MRI	Magnetic Resonance Imaging
CRP	C-reactive Protein
LCR	Lymphocyte–C-reactive Protein Ratio
R0	Microscopically margin-negative surgical resection
